# Editorial: The tumor and microenvironment crosstalk in breast cancer: from biology to therapeutic opportunity

**DOI:** 10.3389/fonc.2023.1339294

**Published:** 2024-01-08

**Authors:** Marta Truffi, Serena Mazzucchelli, Ira Ida Skvortsova

**Affiliations:** ^1^Istituti Clinici Scientifici Maugeri IRCCS, Nanomedicine and Molecular Imaging Laboratory, Pavia, Italy; ^2^Dipartimento di Scienze Biomediche e Cliniche, Università di Milano, Milano, Italy; ^3^EXTRO-Lab, Department of Therapeutic Radiology and Oncology, Innsbruck Medical University, Innsbruck, Austria

**Keywords:** tumor microenvironment, breast cancer, cancer treatment, prognostic biomarkers, immunotherapy

Breast cancer (BC) is a complex process controlled and coordinated by the crosstalk between tumor cells and the several components of the tumor microenvironment (TME), which carry out both pro- and anti-tumor activities in early and advanced settings and play an active role in shaping therapy response. This Research Topic includes eleven papers, four reviews (Wu et al., Song et al., Liu et al., Thu et al.) and seven original articles (Liu et al., Guo et al., Li et al., Zhong et al., Kim et al., Unal et al., Torres-Sanchez et al.), that explore the cellular and molecular players of the breast TME to highlight their pathological implication in BC progression and patient’s outcome. Studying the tumor and microenvironment crosstalk could pave the way toward novel strategies for BC treatment, further supporting the need of more precise tools for personalized therapy planning.

Cancer associated fibroblasts (CAFs) are the most relevant cells in BC TME. Based on cell transcriptome profiling data, Li et al. studied the expression of long noncoding RNAs (lncRNAs) and their regulatory role in TME and immunity, generating a CAF-specific lncRNA (FibLnc) score associated with BC clinical features. From a total of 95 lncRNAs that were found specifically highly expressed in CAFs, 7 were identified to be BC survival-related and were used to construct a survival risk assessment model. The FibLnc score showed good prognostic power in several BC gene expression datasets. The relationship between FibLnc score, mutation status, and drug response was also analyzed, showing that the FibLnc score was able to reflect response to anti-PD-1 or CTLA4 immunotherapy in BC.

Immunotherapy has been frequently coupled with chemotherapy in the treatment of triple negative BC (TNBC), although its use has raised several concerns, since it is a complex and quite expensive treatment. Moreover, only a small portion of patients respond well to these novel therapies, evidencing the need to identify reliable predictive biomarkers and to explore other treatment options. In a retrospective study by Kim et al., a total of 40 samples from metastatic TNBC treated with immune checkpoint inhibitor (ICI) was analyzed for expression of 6 protein markers. The analysis indicated that the lymphocyte-activating gene 3 expression is a predictive biomarker for ICI response. BC with high density of LAG-3^+^CK^+^ cells had worse outcomes with PD-L1/PD-1 inhibitor, whereas BC with high density of LAG-3^+^CK^-^ cells had better outcomes in terms of progression-free survival. Therefore, both tumor-intrinsic and stromal LAG-3 expression are important. In particular, multivariate analysis indicated that tumor LAG-3 was an independent biomarker, suggesting its involvement in driving resistance to PD-1/PD-L1 inhibitors in TNBC. Liu et al., instead, tested the efficacy of compound kushen injection (CKI) as an alternative TME modulator. This is a National Medical Products Administration (NMPA)-approved in China as anticancer agent. It increased chemotherapy efficacy by improving the amount of cytotoxic CD8^+^ T-cells in the tumor, coupled with the T-cells activation and the inhibition of tumor-promoting signaling pathways. Their results support the use of CKI as agent able to potentiate the anti-TNBC effects of chemotherapy.

The crucial role played by TME in immunomodulation was also assessed by Torres-Sanchez et al., which focused their attention on the Rho GTPase Rac and on Cdc42 as molecular targets involved in the crosstalk between cancer and immune cells. Starting from the hypothesis that Rac and Cdc42 inhibitors (Rac/Cdc42i) target also immunosuppressive immune cells, they performed *in vitro* and *in vivo* evaluations demonstrating the effectiveness of Rac/Cdc42i to reduce Rac and Cdc42 activation in macrophages, influencing their cytoskeleton functionality, rather than viability. Indeed, Rac/Cdc42i decreased myeloid cells activation and infiltration into mammary cancers, affecting also IL6 secretion and inducing an antitumor TME by inhibition of metastatic cancer cells, and immunosuppressive myeloid cells.

Since inflammation is the most important feature of TME, the group of Guo et al. analyzed the gene expression of 160 samples of TNBC in comparison to normal tissue samples, with the aim to define a signature of inflammation-related genes (IRGs) able to predict prognosis and treatment response. Thanks to the identification of clusters of IRGs, they developed and validated a prognostic signature that was integrated with clinical data to develop a model for prognosis prediction. High and low risk populations were identified and assessed for their deregulated genes and pathways, and for their IC50 values of chemotherapy and targeted therapy, evidencing how low-risk TNBC were also more sensitive to treatments.

Not only the presence of IRGs signature, but also the presence of a specific microbiota could be a biomarker for BC diagnosis and prognosis. Thu et al. proposed a systematic revision of the literature and a meta-analysis in the attempt to study: (i) microbiota alterations in BC patients, (ii) the impact of treatments on microbial modification and (iii) the impact of microbiome patterns on BC patients receiving the same treatment. They identified some bacterial species elevated in BC patients, despite a low intestinal microbial diversity, evidencing the presence of a complex network that links microbiome, BC and treatment options that required further studies.

In recent years, the association between programmed cell death pathways and the antitumor immunity in BC progression has drawn attention. As an inflammation-related death process, pyroptosis forms an inflammatory microenvironment and has a double-edged impact on both boosting and restraining tumor growth. The crucial factors in the pyroptosis pathways were reviewed by Wu et al. and include inflammatory cytokines (IL-1b and IL-18), inflammasomes (NLRPC4, NLRP3, NLRP1, AIM2), and gasdermins (GSDMA/C/D/E). It has been shown that pyroptosis takes part in the initiation and progression of immune response in BC through interaction with tumor-associated macrophages, myeloid-derived suppressor cells (MDSC), T lymphocytes, dendritic cells and natural killer cells, which promote metastasis, invasion, and angiogenesis in BC. At the same time, active gasdermins delivered to BC cells may destroy tumor cells and enhance immunotherapy. Further understanding of pyroptosis and its functions in BC may provide hints for more actionable targets and candidate drugs to augment immunotherapy efficiency in BC. Another programmed cell death process closely related to BC development is ferroptosis. BC cells exhibit vulnerability to ferroptosis. Ferroptosis is also involved in the regulation of immune microenvironment and immunotherapy resistance in cancer. By using machine learning approaches, a prognostic signature composed of ferroptosis-related genes and hypoxia-related genes (HFRS) was constructed (Zhong et al.). The HFRS was trained on The Cancer Genome Atlas (TGCS) BC cohort and validated on the Molecular Taxonomy of Breast Cancer International Consortium (METABRIC) BC cohort to predict overall survival in BC patients. The high- and low-HFRS patients showed differences in tumor immune cell infiltration, with the high-HFRS group more associated with reduced anti-tumor immunity. Unlike pyroptosis and ferroptosis, cuproptosis is a non-apoptotic programmed cell death pathway induced by the accumulation of intracellular copper. A recent study analyzed the association between cuproptosis and the immune microenvironment in BC (Song et al.). The expression profile of 12 cuproptosis-related genes (CRG) was assessed to construct a CRG signature with prognostic significance. Performing the unsupervised clustering algorithm, BC patients were classified into two cuproptosis patterns (Cluster A and Cluster B), where Cluster B showed more advanced clinicopathological characteristics, worse overall survival and enrichment in most immune cells and important immune checkpoints. Furthermore, the TME characteristics differed significantly in the high- and low-CRG_score groups, suggesting CRGs should be explored to tailor personalized immunotherapy in BC patients.

The crosstalk between BC cells and tissue microenvironment has finally emerged as a crucial mechanism that dictates the formation of metastases in distant organs. The microenvironment factors involved in the formation of liver metastasis and their interaction with BC cells were recently reviewed by Liu et al. Liver sinusoidal endothelial cells, hepatocytes, M2-polarized macrophages, Kupffer cells, CAFs, hepatic stellate cells, neutrophils, MDSC and regulatory T cells are involved in one or multiple phases of BC liver metastasis by direct interaction with BC cells to regulate extravasation and tumor cell seeding, or by releasing cytokines, growth factors, proteases, reactive oxygen species, and recruiting inflammatory cells, thus forming the premetastatic niche, favoring angiogenesis in the micrometastasis and inducing immune tolerance. To date, there are a few studies exploring novel interventional agents targeting the key signaling proteins in the hepatic microenvironment as potential treatment option for BC liver metastasis. Interesting examples are Bafetinib, which blocks the tumor-hepatocytes interaction, and PLD inhibitors, which reduce tumor-promoting macrophages and neutrophil infiltration in primary BC and liver metastasis.

In conclusion, this Research Topic highlights recent advances in the understanding of the pathological implications of the TME in BC progression, suggesting novel prognostic markers and potential therapeutic targets that deserve particular attention to tie these findings to clinical relevance.

## Author contributions

MT: Conceptualization, Supervision, Writing – original draft, Writing – review & editing. SM: Writing – original draft, Writing – review & editing. IS: Writing – original draft, Writing – review & editing.

